# A Comparative Analysis of Hydration Status Among Urban and Rural Adults in Selangor, Malaysia

**DOI:** 10.7759/cureus.87064

**Published:** 2025-06-30

**Authors:** Fazirah Samah, Su Peng Loh, Norhasmah Sulaiman, Wan Ying Gan, Salma Faeza Ahmad Fuzi

**Affiliations:** 1 Department of Nutrition, Universiti Putra Malaysia, Serdang, MYS

**Keywords:** blood measurements, hydration status, malaysia, urban and rural adults, urine tests

## Abstract

Background and aim: Adequate hydration is essential for overall health; however, data on hydration status among Malaysian adults are limited. This study aimed to compare the hydration status of urban and rural adults in Selangor, Malaysia.

Methods: A cross-sectional study was conducted involving participants from urban (n=189) and rural (n=188) areas near Universiti Putra Malaysia (UPM), selected through purposive sampling. The study included 377 healthy adults aged 18-59 years (mean {SD}: 31.34 {11.64} years). Participants with health conditions, such as diabetes, hypertension, gastrointestinal diseases, kidney disorders, and cardiovascular issues, were excluded. Additionally, individuals taking prescription medications, food supplements, consuming high levels of caffeine or alcohol, smoking heavily, or engaging in intense aerobic training were also excluded. Sociodemographic data were collected, and hydration status was assessed using urine samples (first-morning and 24-hour) and blood tests (plasma osmolality, blood count, and renal function). Urine measurements included specific gravity (USG), osmolality (UOSM), volume (UVOL), and colour (UCOL), which were compared against hydration biomarkers. Chi-square tests and independent t-tests (p<0.05) were employed to compare sociodemographic characteristics and hydration status between urban and rural adults. Factor analysis was used to assess the reliability of hydration measurements.

Results: Significant differences were observed in USG, UOSM, and UVOL of first-morning urine samples (χ²{2}=6.537, p=0.038; χ²{1}=6.465, p=0.011; t{375}=-2.10, p=0.036). Additionally, significant differences in USG and UOSM of 24-hour urine samples were noted between urban and rural adults (χ²{2}=27.686, p=0.000; χ²{2}=11.030, p=0.000). Blood tests revealed significant differences in hematocrit (HCT) (t{375}=-1.987, p=0.048), sodium concentration (Na) (t{375}=-4.468, p=0.000), potassium concentration (K) (t{375}=3.563, p=0.000), and estimated glomerular filtration rate (eGFR) (t{371.96}=-4.247, p=0.000) between urban and rural adults. The results indicated that rural adults are less hydrated than their urban counterparts. Factor analysis confirmed that urine measurements are strong indicators of hydration status (quality scores >1), with USG and UOSM in first-morning urine (FMU) showing a strong positive correlation with 24-hour urine samples (0.999), indicating that urine tests are more reliable than blood tests for assessing hydration status.

Conclusion: This study found significant differences in hydration status between urban and rural adults in Selangor, Malaysia, based on both urine and blood measurements; however, urine tests proved to be more reliable for assessing hydration levels. These findings may inform strategies to improve hydration among Malaysian adults. Further research is warranted to investigate factors influencing hydration status.

## Introduction

Water plays a critical role in supporting bodily functions as it constitutes the primary component of cells, tissues, and organs while facilitating the transportation of nutrients and waste products throughout the body [1]. The balance between water intake and loss is reflected in an individual's hydration status. Hydration can be categorized into the following three states: euhydration (normal water content), hyperhydration (excess water), and dehydration (water deficiency) [2]. Maintaining adequate hydration levels is essential for health. Numerous methods exist for assessing hydration status across different settings [3]. The primary methods for assessing hydration include urinary measurements and blood tests. However, evaluating personal hydration requires technical expertise and specialized instruments that must be precise, safe, and affordable. Therefore, selecting appropriate research tools for evaluating water intake and hydration status is crucial. Water consumption levels vary based on population settlements, urban versus rural areas, where rural communities often lack adequate water facilities. In Malaysia, rapid urbanization has led to increased demand for water resources amid a backdrop of population growth [4]. According to the Voluntary National Review (VNR) Report of Malaysia 2021, while access to clean water is high nationally at 95.9%, disparities exist with only 84.7% access in rural areas compared to 98.7% in urban settings [5]. Despite the country's abundant raw water sources from high annual rainfall, a consistent water supply remains a challenge due to various socioeconomic factors. This study aimed to compare the hydration status of urban versus rural adults in Selangor while investigating the reliability of urine versus blood measurements in determining hydration status.

While the importance of adequate hydration is well recognized, there is a noticeable lack of published research on the hydration status of Malaysian adults. Much of the existing literature comes from temperate countries, such as the United States, Canada, and New Zealand, making it less applicable to Malaysia’s hot and humid climate. For instance, Tung et al. reported that 59.6% of Malaysian adolescents were dehydrated, while only 33.0% were well-hydrated; however, studies focusing on adults remain limited [6]. Although some research has examined high-risk groups (elderly, children, athletes, and outdoor workers), adult populations are still underrepresented. Shahar et al. found that lower socioeconomic status (SES) was more prevalent in rural areas, highlighting demographic disparities [7]. Additionally, many local studies rely on self-reported water intake, which is prone to recall bias and may not accurately reflect actual hydration levels. For example, Teng et al. assessed plain water and beverage consumption through a self-administered questionnaire but did not include any objective physiological measures [8]. These gaps underscore the need for more comprehensive, biomarker-based, and demographically inclusive research on hydration in the Malaysian adult population. Therefore, this study aimed to compare the hydration status of urban versus rural adults in Selangor, Malaysia, and hypothesized that adults living in rural areas are more likely to experience dehydration due to inadequate access to potable water. This study is also investigating which markers (urine or blood measurements) represent an accurate indication of overall hydration status.

## Materials and methods

Subjects

This study enrolled healthy Malaysian men and women aged 18-59 years. The inclusion criteria required participants to be Malaysian citizens residing in Selangor and living within a maximum travel time of 30-45 minutes from the investigating center (UPM), applicable to both urban and rural subjects.

Exclusion criteria included the following health conditions: diabetes mellitus, hypertension, gastrointestinal diseases, kidney disorders, and cardiovascular disorders. Additionally, individuals were excluded if they were using prescription medications, such as hypotensive agents, diuretics, or treatments for blood pressure, heart disease, or gastrointestinal issues (e.g., diarrhea or vomiting). Participants who consumed food supplements - including vitamin D, sodium, calcium, detox teas, and sports drinks - were also excluded. Other exclusion factors included caffeine intake of five or more cups per day, alcohol consumption of two or more glasses per day for women and three or more glasses per day for men, smoking 15 or more cigarettes daily, and engaging in aerobic endurance training (such as brisk walking, swimming, running, or cycling) for seven or more hours per week. All these exclusion criteria were crucial for minimizing physiological variability and avoiding confounding influences that could disrupt fluid balance, kidney function, and hydration biomarkers. By doing so, the study ensured a more accurate and valid interpretation of hydration level, specifically among Malaysian adults.

Study design, sampling, and location

This cross-sectional study was conducted in Selangor, employing a two-stage sampling method. First, convenience sampling was used to identify urban and rural localities within 30-47 minutes of UPM. The urban and rural areas were identified using a list obtained from the Department of the Director General of Lands and Mines [9]. Secondly, purposive sampling was used to select specific study locations within these areas, namely Petaling (urban) and Hulu Langat (rural).

Study approval

Approval for this study was obtained from the Ethics Committee for Research Involving Human Subjects at Universiti Putra Malaysia (JKEUPM) (date of approval: July 13, 2022, and August 21, 2023; ref no: 2022-163). The approval letter, letter of application permission, study proposal, and brochure/poster to carry out data collection have been sent to the representative management officer (Jawatankuasa Rukun Tetangga or Ketua Kampung) in selected residential areas in both urban and rural areas. These documents were intended to inform the local authorities about the study objectives, procedures, and ethical compliance and to obtain official permission to proceed with participant recruitment and data collection within the communities. This step ensured transparency, fostered cooperation, and upheld institutional and community engagement standards.

Sample size calculation

The sample size was determined using the two population proportions formula by Lachenbruch and Clements [10].

$n = \frac{(Z_{1-\alpha/2} + Z_{1-\beta})^2 \left[ p_1(1 - p_1) + p_2(1 - p_2) \right]}{(p_1 - p_2)^2}$, where $Z_{1 - \alpha/2}$ represents the 95% confidence level, $Z_{1 - \beta}$ denotes 80% power, p is the average proportion between groups $p = \frac{p_1 + p_2}{2}$, p_1_ is the proportion of adults with adequate plain water intake in urban (50.5%) versus rural (45.7%) areas, and p_2_ is the proportion with inadequate intake in urban (24.9%) versus rural (28.6%) areas, based on NHMS 2019 data.

The sample size of this study was 253 (urban) and 470 (rural) participants (median {n} = 361.5) in total. To compensate for missing data or non-response in the study, an additional 20% of the calculated sample size has been added, resulting in a total of 400 participants required for the study. This adjustment is designed to adhere to best practices in research methodology, considering the possibility of participant attrition. By anticipating potential dropouts, this approach ensures the study retains robust statistical power and the integrity of its data, thereby safeguarding the reliability of the research outcomes.

Research instruments

A set of questionnaires, written in both English and Bahasa Malaysia, was used to determine the independent and dependent variables of the study. It consisted of three parts as follows: sociodemographic characteristics (10 questions), urine assessment (four questions), and blood assessment (six questions). The independent variables (IV) were sociodemographic characteristics, while the dependent variables (DV) were urine and blood assessment for hydration status.

Sociodemographics characteristics

The participants were interviewed using a self-constructed questionnaire to gather information about their age, gender, race, residential area (urban or rural), religion, marital status, educational level, employment status, and monthly household income.

Urine assessment

Urinary hydration assessment included measurements of urine specific gravity (USG), urine osmolality (UOSM), urine color (UCOL), and urine volume (UVOL). Participants’ urine samples were collected at the following two time points: the first-morning void (FMU) and over a 24-hour period (24hU), representing total daily urine output. Both FMU and 24hU were collected separately and analyzed for hydration status. For the 24hU collection, participants were instructed to collect all urine voids over a full 24-hour period. The procedure began by discarding the FMU on day one and subsequently collecting all urine voided throughout the day and night, including the FMU on day two. Participants were provided with labelled containers and detailed instructions to ensure accurate and complete sample collection. This method allows for a comprehensive assessment of urinary excretion over a 24-hour period.

For urine analysis, the UOSM prediction formula by Imran et al. was used [11].

UOSM = 100 × (-183.22 + 181.63 × SG + 0.42 × pH + 0.57 × glucose + 0.57 × ketones - 0.49 × bilirubin + 0.30 × urobilinogen - 0.33 × protein)

USG was determined by comparing the value to the dipstick urinalysis chart, while UCOL was assessed by comparing the urine sample's color in a clear 15-mL glass tube against a white background under fluorescent lighting, using an eight-color scale ranging from very pale yellow to brownish green [12]. Lighter urine color indicates more water excretion, while darker urine color indicates less. The results were compared to the reference values of hydration biomarkers as provided by Armstrong et al. (Table 1) [13].

**Table 1:** Reference values for urine hydration biomarkers.

Hydration category	First-morning urine (FMU)		24-hour urine (24hU)		
	Urine specific gravity (USG)	Urine osmolality (UOSM/kg)	Urine specific gravity (USG)	Urine osmolality (UOSM/kg)	Urine volume (mL)
Hyperhydrated	<1.017 to 1.021	<545 to 713	<1.012 to 1.014	<377 to 475	>2250 to 1898
Euhydrated	1.022 to 1.026	714 to 924	1.015 to 1.020	476 to 766	1897 to 1226
Dehydrated	1.027 to >1.031	925 to >1129	1.021 to >1.027	767 to >1013	1225 to <875

Blood tests

Blood hydration assessment included plasma osmolality, full blood count (hemoglobin and hematocrit levels), and renal profile tests (sodium, potassium, urea, creatinine, chloride, uric acid, and estimated glomerular filtration rate {eGFR}) [14]. A certified and trained phlebotomist, using safe and sterile techniques and laboratory equipment, was required for these measurements. Subjects maintained their normal lifestyles and were not required to fast before data collection. The blood collection and assessment procedure from Alberta Health Services (AHS) Laboratory Services was adapted for this study [15]. Hydration cut-off points for blood tests are shown in Table 2 [15]. Sample analysis was conducted at the Faculty of Medicine and Health Sciences (FMHS), UPM, and Innoquest Pathology Sdn. Bhd.

**Table 2:** Reference values for blood hydration biomarkers. eGFR: estimated glomerular filtration rate

Variables	Interpretation
Plasma osmolality (POSM)	Hyperhydrated: <278 mOsm/kg; euhydrated: 278 to 298 mOsm/kg; dehydrated: >298 mOsm/kg
Hemoglobin	Male: 13.0 to 18.0 g/dL; female: 12.0 to 15.0 g/dL
Hematocrit	Male: 40 to 55%; female: 36 to 46%
Sodium concentration (mEq/L)	135 to 145
Potassium concentration (mEq/L)	3.5 to 5.1
Urea (mmol/L)	2.5 to 8.0
Creatinine (mmol/L)	Male: 50 to 116; female: 40 to 80
eGFR (mL/min/1.73 m^2^)	≥90
Chloride (mmol/L)	95 to 110
Uric acid (mmol/L)	Male: 0.18 to 0.47; female: 0.15 to 0.45

Data collection procedure

Pre-testing

Ten subjects were selected for a pre-test session to evaluate the study's instruments. Those who did not meet the inclusion criteria were excluded. These participants were selected to represent the target population. The pre-test involved administering the questionnaires to assess completion time, clarity of instructions, and overall instrument suitability. Participants in the pre-test were not included in the final study sample. Based on the pre-test results, the content was adjusted and modified for better understanding.

Subject Recruitment

Data collection took place from November 2022 to August 2023. Meetings were held with the resident association in the urban area and the neighborhood committee in the rural area to obtain permission and discuss the data collection process. A total of 350 subjects who met the selection criteria were invited to participate in the study. Notices, including posters and brochures, were distributed one week before data collection to inform eligible residents. Data collection was conducted at the community hall. During the session, subjects received an information sheet outlining the risks, benefits, time commitment, and procedures, along with a consent form. Each subject underwent the study measurements, with each session lasting about 30 minutes. A token of appreciation (RM50) was given to each subject at the end of their session.

Statistical analysis

The IBM Statistical Package for Social Sciences (SPSS) 25.0 (Armonk, NY: IBM Corp.) was used for data analysis. Normal distribution assumptions for each variable were evaluated, with a distribution considered approximately normal if skewness and kurtosis values ranged from -1 to 1, and the Shapiro-Wilk test had a p-value greater than 0.05. If normality was not met, the Mann-Whitney U test was used, and results were reported using medians and interquartile ranges (Q1-Q3). The chi-square test was used to compare the sociodemographic characteristics and hydration status of urban and rural adults (p<0.05). For some sociodemographic variables with expected cell counts less than five, Fisher’s exact test was used instead. Next, an independent t-test was used to compare urine and blood test results between urban and rural adults (p<0.05). Principal component analysis (PCA) of factor analysis (eigenvalues >1) was then applied to identify which markers (urine or blood test) best represent hydration status.

## Results

Sociodemographic characteristics among urban and rural adults in Selangor, Malaysia

A total of 390 adults participated in this study. However, due to withdrawals (7) and loss to follow-up (6), the final number of completed participants was 377 (urban=189 and rural=188). A chi-square test of independence was conducted to examine the association between sociodemographic characteristics and residential areas (urban and rural) (Table 3). There was significant difference in age category (χ²{2}=91.800; p<0.001), races (χ²{3}=39.856; p<0.001), religions (χ²{3}=40.285; p<0.001), marital status (χ²{3}=102.110; p<0.001), employment status (χ²{5}=112.883; p<0.001), and household income classification (χ²{1}=30.382; p<0.001) between urban and rural adults Selangor, Malaysia. However, the assumptions of the chi-square test were violated for several factors (races, religions, marital status, and employment status) as there were cells having expected counts less than five. Given this violation, Fisher’s exact test was used as a more appropriate alternative. The result from Fisher’s exact test also indicated a statistically significant association between these variables (p<0.001).

**Table 3:** Sociodemographic characteristics among urban and rural adults in Selangor, Malaysia. *P-value <0.05 was statistically significant.

Factors	N (%)		Chi-square test χ² (df)	Fisher’s exact test	p-Value
	Urban (n=189)	Rural (n=188)			
Gender			0.469 (1)	-	0.494
Male	50 (26.5)	44 (23.4)			
Female	139 (73.5)	144 (76.6)			
Age category (years)			91.800 (2)	-	<0.001*
Young adults (18-29)	52 (27.5)	144 (76.6)			
Early-middle adults (30-44)	90 (47.6)	25 (13.3)			
Late-middle adults (45-59)	47 (24.9)	19 (10.1)			
Races			39.856 (3)	43.215	<0.001*
Malay	177 (93.7)	132 (70.2)			
Chinese	7 (3.7)	50 (26.6)			
Indian	3 (1.6)	2 (1.1)			
Others	2 (1.1)	4 (2.1)			
Religions			40.285 (3)	43.393	<0.001*
Islam	177 (93.7)	133 (70.7)			
Buddhism	6 (3.2)	46 (24.5)			
Hindu	3 (1.6)	1 (0.5)			
Christian	3 (1.6)	8 (4.3)			
Marital status			102.110 (3)	106.212	<0.001*
Single	53 (28.0)	148 (78.7)			
Married	133 (70.4)	37 (19.7)			
Widower (male)	0 (0.0)	2 (1.1)			
Widower (female)	3 (1.6)	1 (0.5)			
Educational level			8.396 (5)	8.620	0.136
No formal education	0 (0.0)	0 (0.0)			
Primary school (UPSR)	0 (0.0)	0 (0.0)			
Secondary school (LCE/SRP/PMR/PT3)	1 (0.5)	1 (0.5)			
Upper secondary school (SPM/O-level)	24 (12.7)	17 (9.0)			
HSC/STPM/STAM/A-level/certificate/diploma	24 (12.7)	36 (19.1)			
Bachelor degree	129 (68.3)	131 (69.7)			
Master degree	9 (4.8)	2 (1.1)			
PhD	2 (1.1)	1 (0.5)			
Type of employment			112.883 (5)	117.844	<0.001*
Civil servants	132 (69.8)	36 (19.1)			
Private sector workers	8 (4.2)	9 (4.8)			
Government pensioner	2 (1.1)	0 (0.0)			
Private sector retirees	3 (1.6)	0 (0.0)			
Self-employed	42 (23.1)	140 (74.5)			
Housewife	2 (1.1)	3 (1.6)			
Unemployed	0 (0.0)	0 (0.0)			
Others	0 (0.0)	0 (0.0)			
Household income classification			30.382 (1)	-	<0.001*
B40 (RM0 - RM4849)	69 (36.5)	122 (64.9)			
M40 (RM4850 - RM10 959)	120 (63.5)	66 (35.1)			
T20 (RM10 960 - more than RM15 040)	0 (0.0)	0 (0.0)			

Most of the urban population was in the early-middle adults age category (30-44 years) (n {%}=90 {47.6}), Malay (n {%}=177 {93.7}), Islam (n {%}=177 {93.7}), married (n {%}=133 {70.4}), had a bachelor’s degree (n {%}=129 {68.3}), worked as civil servants (n {%}=132 {69.8}), and was in the M40 household income classification (n {%}=120 {63.5}). The majority of the rural population were young adults aged 18-29 years (n=144, 76.6%), Malay (n=132, 70.2%), Muslim (n=133, 70.7%), single (n=148, 78.7%), held a bachelor's degree (n=131, 69.7%), were self-employed (n=140, 74.5%), and belonged to the B40 household income group (n=122, 64.9%).

Comparison of hydration status among urban and rural adults in Selangor, Malaysia

**Figure 1:**
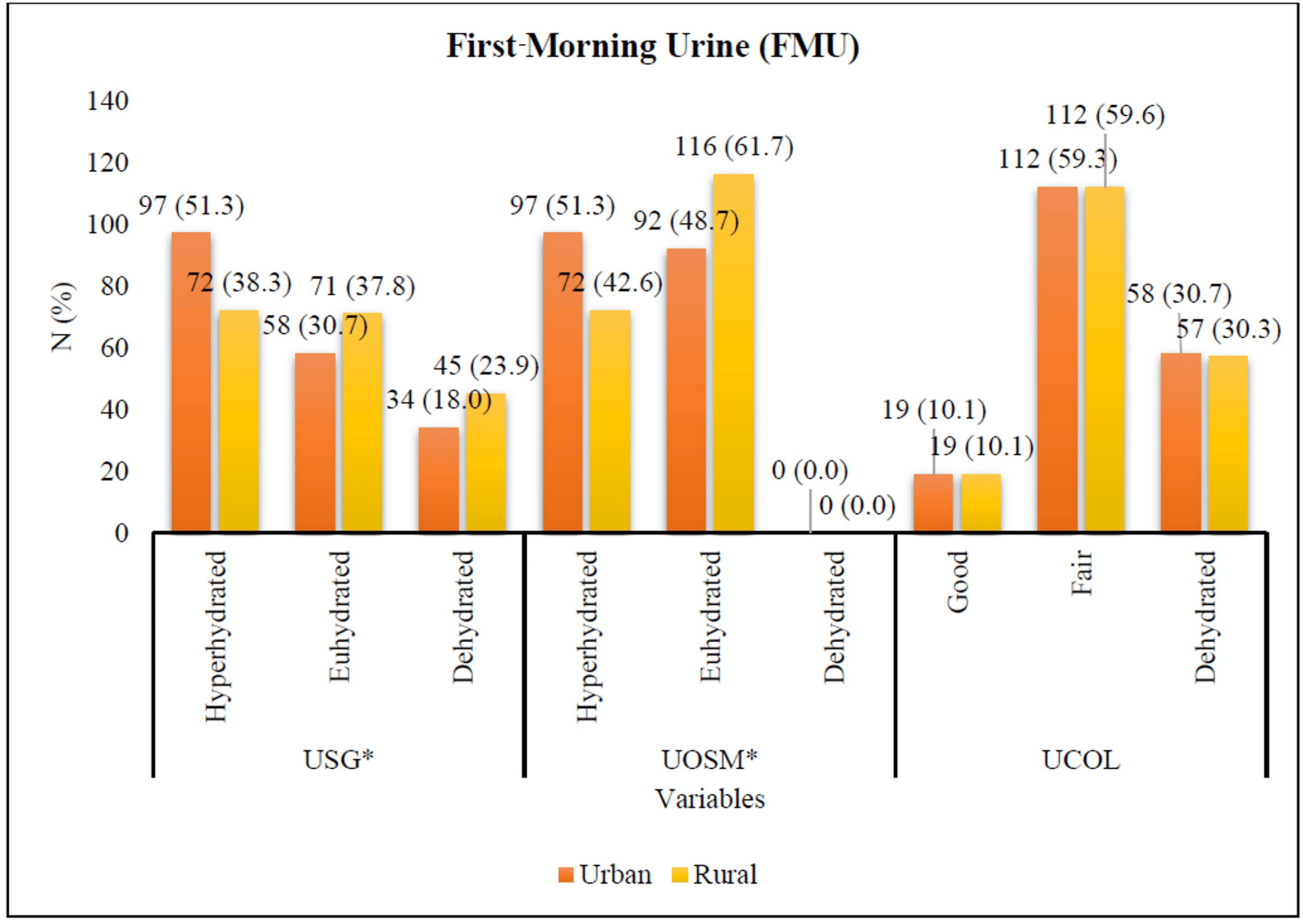
USG, UOSM, and UCOL of FMU differences between urban and rural adults in Selangor, Malaysia. *P-value <0.05 was statistically significant. FMU: first-morning urine; USG: urine specific gravity; UOSM: urine osmolality; UCOL: urine color; N: number

**Figure 2:**
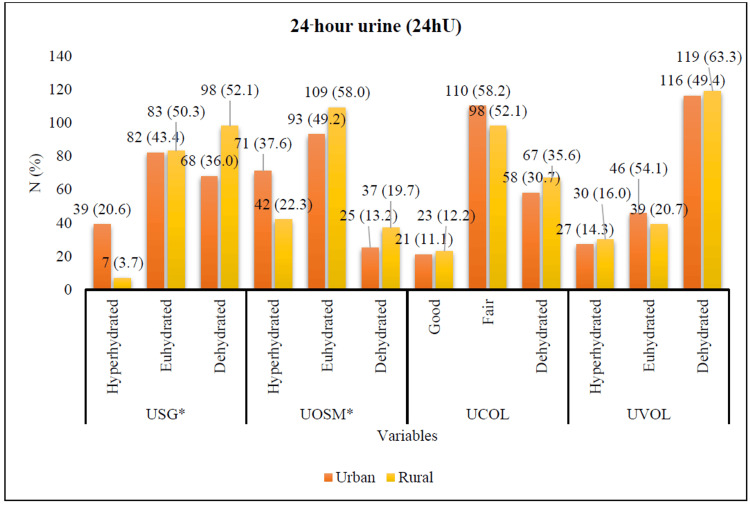
USG, UOSM, UCOL, and UVOL of 24hU differences between urban and rural adults in Selangor, Malaysia. *P-value <0.05 was statistically significant. 24hU: 24-hour urine; USG: urine specific gravity; UOSM: urine osmolality; UCOL: urine color; UVOL: urine volume; N: number

Figures 1, 2 show the differences in hydration status between urban and rural adults based on first-morning urine (FMU) and 24-hour urine (24hU) measurements. Figure 1 shows significant differences in USG (χ²{2}=6.537; p=0.038), UOSM (χ²{1}=6.465; p=0.011) of FMU between urban and rural adults in Selangor, Malaysia. Among urban adults, a higher proportion exhibited hyperhydration based on USG (n {%}=97 {51.3}) and UOSM (n {%}=97 {51.3}), with the majority having fair urine color (n {%}=112 {59.3}). In contrast, among rural adults, 72 individuals (38.3%) were classified as hyperhydrated based on USG, while 116 (61.7%) were euhydrated according to UOSM, and 112 (59.6%) had fair urine color. Additionally, rural adults had a higher mean urine volume (mean=43.45, SD=10.63) compared to their urban counterparts (mean=41.22, SD=10.03). Overall, based on FMU measurements, both urban and rural adults demonstrated adequate hydration status.

According to Figure 2, there were significant differences in USG (χ² {2}=27.686; p=0.000) and UOSM (χ² {2}=11.030; p=0.004) of 24hU between urban and rural adults in Selangor, Malaysia. Among urban adults, the majority were classified as euhydrated based on USG (n {%}=82 {43.4}) and UOSM (n {%}=93 {49.2}), with most exhibiting fair urine color (n {%}=110 {58.2}). However, a considerable proportion of urban adults presented with low urine volume, indicating dehydration (n {%}=116 {49.4}). In contrast, among rural adults, more than half were categorized as dehydrated based on USG (n {%}=98 {52.1}), while a higher percentage remained euhydrated according to UOSM (n {%}=109 {58.0}). Similarly, 98 (52.1%) rural participants had fair urine color, yet a substantial proportion exhibited dehydration based on urine volume (n {%}=119 {63.3%}). Overall, 24-hour urine measurements indicated that urban adults maintained adequate hydration, whereas rural adults showed signs of dehydration, particularly in terms of urine volume and specific gravity.

Table 4 compares the hydration status of urban and rural adults based on urine volume from both FMU and 24hU, as well as blood measurements. There was significant difference found in urine volume of FMU (t {375}=-2.10; p=0.036), hematocrit (t {375}=-1.987; p=0.048), sodium concentration (t {375}=-4.468; p=0.000), potassium concentration (t {375}=3.563; p=0.000), and estimated glomerular filtration rate (eGFR) (t {371.96}=-4.247; p=0.000) between urban and rural adults in Selangor, Malaysia. Rural adults had a higher mean hematocrit, sodium concentration, and eGFR, while a lower mean potassium compared to urban adults. Overall, both urban and rural adults had a normal mean range for all blood tests of hydration status.

**Table 4:** An independent t-test on the comparison of hydration status among urban and rural adults based on urine volume (FMU) and blood measurements. *P-value <0.05 was statistically significant. eGFR: estimated glomerular filtration rate

Factors	Mean±SD		Levene’s test		t (df)	p-Value	Mean difference	95% CI (lower, upper)
	Urban (n=189)	Rural (n=188)	F-value	p-Value				
Urine volume (FMU)	41.22±10.03	43.45±10.63	0.530	0.467	-2.10 (375)	0.036*	-2.235	-4.328, -0.142
Serum osmolality	294.42±7.892	295.63±33.178	0.780	0.378	-0.488 (375)	0.626	-1.210	-6.088, 3.669
Hemoglobin	129.07±16.965	130.37±16.190	0.017	0.896	-0.760 (375)	0.448	-1.298	-4.657, 2.061
Hematocrit (PCV)	0.41±0.050	0.42±0.045	0.255	0.614	-1.987 (375)	0.048*	-0.010	-0.019, -0.001
Sodium concentration	139.35±2.280	140.53±2.810	2.535	0.112	-4.468 (375)	<0.001*	-1.177	-1.696, -0.659
Potassium concentration	4.36±0.443	4.21±0.394	0.652	0.420	3.563 (375)	<0.001*	0.154	0.069, 0.239
Chloride	100.54±2.712	100.63±3.062	2.682	0.102	-0.295 (375)	0.768	-0.088	-0.674, 0.498
Urea	3.55±0.917	3.43±0.885	2.825	0.094	1.292 (375)	0.197	0.120	-0.063, 0.302
Creatinine	67.26±14.268	66.76±14.781	0.001	0.972	0.337 (375)	0.736	0.504	-2.438, 3.446
eGFR	109.87±13.638	116.11±14.852	4.704	0.031	-4.247 (372)	<0.001*	-6.239	-9.126, -3.351
Uric acid	0.30±0.075	0.29±0.073	0.207	0.649	1.769 (375)	0.078	0.014	-0.002, 0.029

Comparison between hydration measurements (urine versus blood markers)

A principal component analysis (PCA) of factor analysis was conducted to determine the underlying structure of hydration-related variables, which included urine-based (urine osmolality, urine specific gravity, urine volume) and blood markers (plasma osmolality, hemoglobin, hematocrit, sodium concentration, potassium concentration, chloride, urea, creatinine, eGFR, and uric acid). The Kaiser-Meyer-Olkin (KMO) score for sample adequacy was tested before performing PCA, yielding a result of 0.611, which falls within an acceptable range (≥0.60), indicating that the data are suitable for factor analysis. The correlation matrix could be used for PCA because Bartlett's test of sphericity showed that it was significant (χ² {78}=4132.72, p<0.001).

A six-component solution was extracted using eigenvalues greater than 1.0 (Kaiser’s criterion), accounting for 72.91% of the total variance in hydration-related measurements. The first principal component (PC1) had an eigenvalue of 3.683, explained 23.02% of the total variance, and included high loadings for urine specific gravity (FMU and 24hU) and urine osmolality (FMU and 24hU). The second principal component (PC2), which mostly captured blood-based parameters like hemoglobin, hematocrit, and creatinine, explained 17.96% (2.873) of the variance. The remaining elements stood for plasma osmolality (PC5), electrolyte regulation (PC4), kidney function (PC3: eGFR, urea), and urine volume (PC6: urine volume FMU and 24hU). Table 5 presents a summary of the eigenvalues and variance explained by each component.

**Table 5:** Correlation matrix of urine measurements. *P-value <0.05 was statistically significant. USG: urine specific gravity; UOSM: urine osmolality; UVOL: urine volume; FMU: first-morning urine; 24hU: 24-hour urine

Component	USG (FMU)	UOSM (FMU)	UVOL (FMU)	USG (24hU)	UOSM (24hU)	UVOL (24hU)
USG (FMU)	1.000	1.000	-0.100	0.589	0.587	-0.361
UOSM (FMU)	1.000*	1.000	-0.100	0.589	0.587	-0.361
UVOL (FMU)	-0.100	-0.100	1.000	-0.100	-0.099	0.118
USG (24hU)	0.589	0.589	-0.100	1.000	0.999	-0.550
UOSM (24hU)	0.587	0.587	-0.099	0.999*	1.000	-0.550
UVOL (24hU)	-0.361	-0.361	0.118	-0.550*	-0.550*	1.000

**Figure 3:**
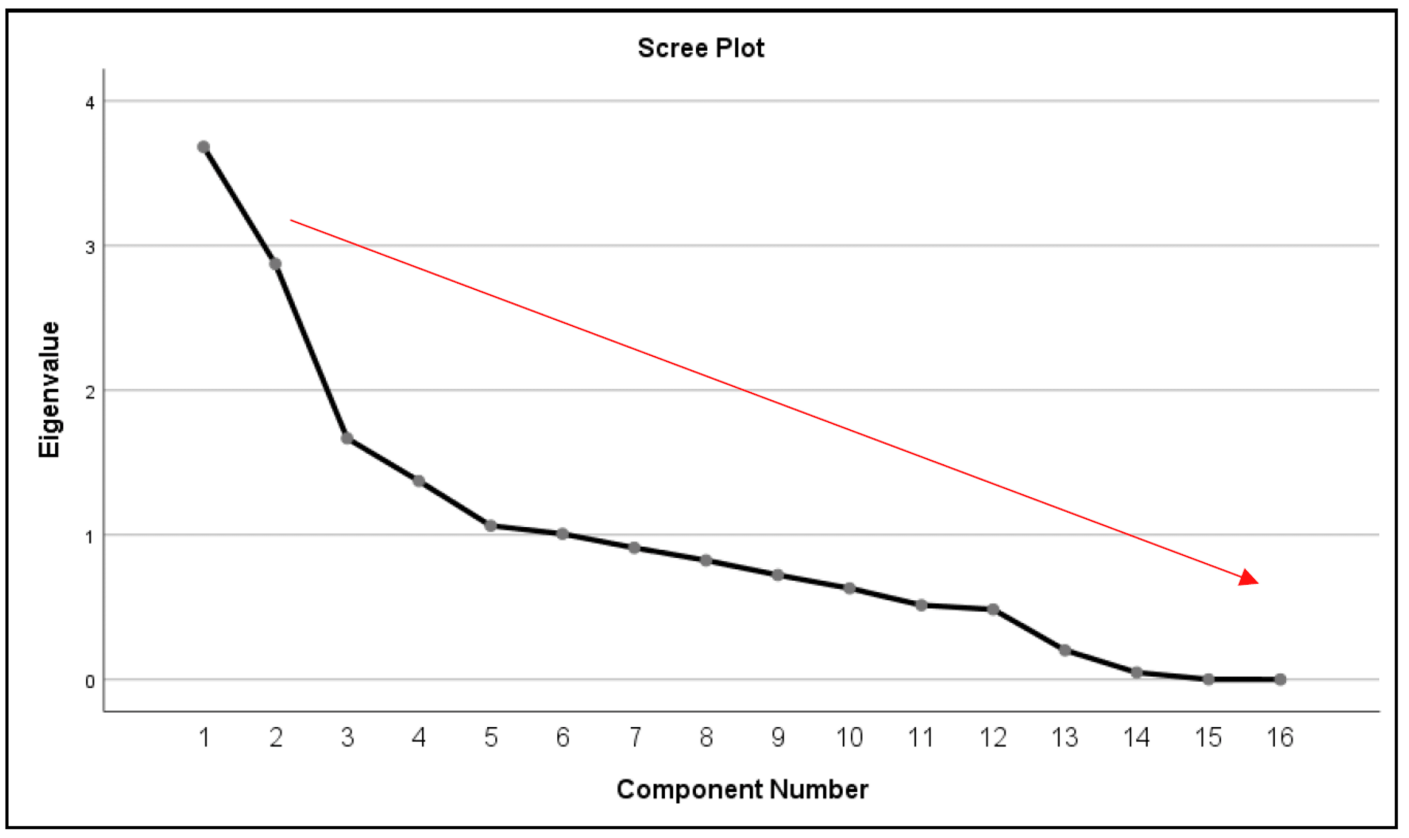
The scree plot graph of hydration measurements.

The scree plot shows a sharp drop in eigenvalues for the first components and flattens around the sixth component (Figure 3). This "elbow" means that six components are worth keeping. Beyond this threshold, the remaining components have eigenvalues less than 1.0, representing noise, and add little value. Strong loadings (≥0.60) for each hydration-related measure were identified using the rotated component matrix, which further revealed the factor structure (Table 6). In general, values close to 1 reflect stronger correlations. The results suggested that urine markers (USG and UOSM) loaded highly on the first principal component, revealing their strong relationship with hydration. On the other hand, blood-derived markers like hemoglobin, hematocrit, creatinine, and plasma osmolality are grouped into individual components. This strongly suggests that USG and UOSM are superior predictors of hydration status compared to blood-derived measurements, which appeared to have a smaller impact.

**Table 6:** Rotated component matrix showing factor loadings. eGFR: estimated glomerular filtration rate; USG: urine specific gravity; FMU: first-morning urine; 24hU: 24-hour urine; PC: principal component; PCV:

Variables	PC1	PC2	PC3	PC4	PC5	PC6
USG (FMU)	0.831	-0.219	0.042	0.087	-0.021	0.067
Urine osmolality (FMU)	0.831	-0.219	0.042	0.087	-0.021	0.067
Urine volume (FMU)	-0.147	0.071	0.157	0.175	-0.382	0.741
USG (24hU)	0.835	-0.328	-0.085	-0.119	0.013	0.089
Urine osmolality (24hU)	0.834	-0.328	-0.085	-0.120	0.013	0.090
Urine volume (24hU)	-0.556	0.349	0.094	0.066	0.064	0.129
Plasma osmolality	-0.027	0.006	-0.131	-0.097	0.873	0.129
Hemoglobin	0.373	0.730	0.410	-0.206	0.023	-0.066
Hematocrit (PCV)	0.402	0.684	0.496	-0.171	0.029	-0.045
Sodium	0.169	0.060	0.468	0.672	0.167	-0.161
Potassium	0.099	0.112	-0.455	0.067	-0.310	-0.492
Chloride	-0.032	-0.305	0.199	0.751	0.047	-0.112
Urea	0.170	0.287	-0.551	0.328	0.067	0.071
Creatinine	0.373	0.751	-0.150	0.212	0.013	0.134
eGFR	-0.118	-0.532	0.625	-0.198	-0.075	-0.210
Uric acid	0.232	0.630	-0.051	0.023	-0.107	-0.212

## Discussion

Malaysia's rapid economic growth and rising living costs have significantly impacted the socioeconomic status of its population. This study reveals that rural populations have lower incomes compared to their urban counterparts, in which there were 122 (64.9%) rural participants compared to 69 (36.5%) urban participants in the B40 household income classification, aligned with the Department of Statistics Malaysia's 2016 report. The report indicated that rural household incomes consistently lag urban incomes and constitute a higher proportion of the bottom 40% in income classification [16]. This rural-urban disparity, exacerbated by urbanization, is further reflected in Malaysia's high national Gini coefficient - 43.9% for urban areas versus 42.6% for rural areas - indicating substantial income inequality [17]. Living in rural households increases the likelihood of poverty, which is characterized by social exclusion and limited access to essential services, such as education, healthcare, nutrition, housing, water supply, and sanitation [18]. These challenges are particularly prevalent in rural communities, where employment opportunities are limited, making it difficult for residents to achieve higher incomes. Despite holding bachelor's degrees similar to those of urban adults, many rural residents are self-employed, often with unstable and low incomes. In contrast, urban residents predominantly work as civil servants with fixed salaries and annual raises.

This study is the first to detail hydration biomarkers in healthy adults living in both urban and rural areas of Malaysia, highlighting significant differences in hydration status as measured through urine and blood analyses. These measurements provide insights into water intake, losses, and physiological processes. Notably, significant differences in mean urine osmolality (UOSM) have been documented between urban and rural populations in European countries; for instance, urban Germany reported a higher mean UOSM (860 mOsm/kg) compared to rural Poland (392 mOsm/kg) [19]. Rural residents often experience dehydration due to health and nutrition disparities linked to lower socioeconomic status. These disparities include limited insurance coverage, restricted access to quality healthcare and safe water, as well as regional dietary habits [7]. A study by Brooks et al. found that adults with lower incomes had a 20% higher likelihood of inadequate hydration compared to their higher-income counterparts [20]. The financial burden of purchasing beverages - including bottled water - exacerbates this issue for lower-income rural populations. Additionally, rural adults frequently face challenges in accessing safe water and affordable healthcare due to geographic isolation and increasing medical expenses [21]. Such disparities in hydration status among the rural population adversely affect their overall health and well-being.

Blood sodium results indicate that the mean blood sodium concentration is higher among rural adults compared to their urban counterparts. High sodium levels in blood disrupt the body’s fluid balance as they raise the osmotic pressure of extracellular fluids, stimulating thirst to maintain equilibrium. In rural areas, there was limited access to water, and often the use of salt for food preservation resulted in not adequately compensating for this imbalance; hence, dehydration occurred. By that, when the individuals consume less water, their blood sodium level might rise.

This study found that urine specific gravity (USG) and urine osmolality (UOSM) are more accurate indicators of hydration status than blood tests. Both USG and UOSM show how well the kidneys concentrate solutes in urine, making them sensitive measures of hydration. Urine tests are also non-invasive, low-cost, and provide quick results, making them useful in both clinical and field settings. Hustrini et al. reported that urine osmolality had 70% sensitivity and 76% specificity in detecting good hydration [22]. Similarly, Baron et al. found that USG and UOSM are suitable for large studies and various settings because urine collection is simple, affordable, and requires minimal technical skill [23]. Therefore, using USG and UOSM can improve the reliability and ease of assessing hydration.

In contrast, plasma osmolality measurements showed a weak correlation with hydration status (eigenvalues=0.91). This is due to the availability of urine indicators that are more sensitive to fluid intake or loss, which are better suited for detecting hydration changes. Additionally, factors such as the condition and timing of blood collection, dietary intake, and environments can further mask variations in plasma osmolality, reducing its correlation with actual hydration status in field or community-based studies [24]. This discrepancy highlights the limitations of plasma osmolality as a hydration indicator alongside its invasive nature and associated costs.

Moreover, the use of plasma osmolality as a hydration marker poses several practical limitations in field settings. It requires invasive blood sampling, necessitating the presence of certified phlebotomists, sterile procedures, and informed consent, all of which can be challenging to implement in large-scale or community-based research. In contrast, urine-based measures, such as USG and UOSM, rely on non-invasive sample collection, making them more acceptable to participants and better suited for diverse populations. Additionally, plasma osmolality analysis depends on advanced laboratory infrastructure and strict sample handling protocols, which are often unavailable or cost-prohibitive outside clinical environments. On the other hand, USG can be assessed with portable, user-friendly refractometers, offering a practical and efficient alternative for real-time hydration monitoring in field-based studies. This highlights the superior feasibility and accessibility of urine indices for hydration assessment in non-clinical settings.

Furthermore, this study identified a positive correlation between USG and UOSM values consistent with previous research findings [25,26]. Both USG and UOSM effectively reflect hydration status since they respond sensitively to changes in fluid intake [27]. This sensitivity stems from the kidneys’ ability to rapidly adjust urine concentration in response to hydration levels. For example, the kidneys excrete more diluted urine, lowering USG and UOSM levels when fluid intake is increased. Conversely, the kidneys conserve water, producing more concentrated urine, reflected in higher USG and UOSM values during dehydration. These changes make USG and UOSM effective for capturing acute shifts in hydration status. In contrast, plasma osmolality is tightly regulated by homeostatic mechanisms, such as the release of antidiuretic hormone (ADH), maintaining plasma osmolality within a narrow range despite fluctuations in hydration [28]. As a result, plasma osmolality may not reflect subtle or short-term changes in fluid balance. Hence, both USG and UOSM differ from other hydration indicators because they provide direct measures of renal function and fluid balance, greater sensitivity to short-term changes in hydration, and non-invasive and practical measurements. Thus, these measurements serve as valuable tools for general hydration monitoring and mitigating health risks.

This study has several strengths, including a balanced representation of participants from both urban and rural settings, thus making the findings applicable across these populations. Additionally, it provides novel insights into hydration levels among Malaysian adults-valuable data that can inform nutritionists, dieticians, and health practitioners aiming to enhance water intake among Malaysians. However, limitations exist as follows: the data cannot be generalized to the entire Malaysian population, as this study was conducted solely within one state. Furthermore, factors influencing hydration status, such as anthropometric measurements, lifestyle choices, and environmental conditions, were not examined. Future research should address these variables.

## Conclusions

The findings indicate significant differences in hydration status between urban and rural adults in Selangor based on various indices, including USG, UOSM, first-morning urine volume, hematocrit levels, sodium concentration, potassium concentration, and estimated glomerular filtration rate (eGFR), which are primarily attributed to socioeconomic factors. Urine tests proved more reliable indicators of hydration status than blood tests. These insights can serve as baseline references for improving hydration among Malaysian adults while underscoring the need for further investigation into factors contributing to these disparities. People were encouraged to stay hydrated through education and nutrition programs that emphasized drinking enough water, eating fresh foods, reducing salt intake, and doing regular urine checks to detect dehydration early. Ensuring access to clean water, especially in rural areas, was also important. These efforts can help narrow the gap in hydration levels between urban and rural communities and improve overall health.
